# Sex-specific reporting patterns and onset timing of immune-related adverse events associated with nivolumab and pembrolizumab: a dual-database pharmacovigilance analysis

**DOI:** 10.3389/fimmu.2026.1837640

**Published:** 2026-05-13

**Authors:** Yu Bai, Hong Liu, Hao Meng

**Affiliations:** 1 Key Laboratory of Carcinogenesis and Translational Research (Ministry of Education), Department of Pharmacy, Peking University Cancer Hospital and Institute, Beijing, China; 2 Department of Cardiovascular Disease, Beijing Hospital of Traditional Chinese Medicine, Capital Medical University, Beijing, China

**Keywords:** food and drug administration adverse event reporting system, immune-related adverse events, Japanese adverse drug event report, pharmacovigilance, programmed death-1 inhibitors (PD-1), sex difference

## Abstract

**Background:**

Nivolumab and pembrolizumab are associated with various immune-related adverse events (irAEs). However, sex-specific differences in irAE reporting patterns remain incompletely characterized, especially regarding timing of onset. Therefore, we conducted a dual-database pharmacovigilance study to evaluate the sex-specific reporting patterns and time-to-onset (TTO) of irAEs associated with nivolumab and pembrolizumab.

**Methods:**

The U.S. Food and Drug Administration Adverse Event Reporting System (FAERS) and Japanese Adverse Drug Event Report (JADER) databases from 2014 to 2025 were used for the analyses. Eligible reports included those in which sex of a patient was known and nivolumab or pembrolizumab was recorded as a suspected drug. Sex-stratified disproportionality signals were assessed using multivariable logistic regression to estimate the adjusted reporting odds ratios, and sex differences in onset timing were evaluated using multivariable Weibull accelerated failure time models to estimate the adjusted time ratios.

**Results:**

In total, 146,796 eligible reports from FAERS and 56,767 from JADER were included. At the System Organ Class level, endocrine disorders showed consistent female-predominant reporting signals in both databases, whereas respiratory, thoracic, and mediastinal disorders showed consistent male-predominant patterns in both databases. At the individual irAE level, hypothyroidism and hyperthyroidism were the most prominent female-predominant signals, whereas interstitial lung disease was the most prominent male-predominant signal. TTO analysis results showed that endocrine disorders, especially hypothyroidism, occurred earlier in females than in males in both databases.

**Conclusions:**

This study identified sex-associated patterns in endocrine and pulmonary irAEs related to nivolumab and pembrolizumab. However, our findings should be interpreted as signal-generating rather than causal, as they suggest that sex may be a clinically relevant factor in refining toxicity surveillance and individualized safety management during PD-1 inhibitor therapy.

## 1 Introduction

Immune checkpoint inhibitors (ICIs), particularly antibodies targeting programmed death-1 (PD-1), have reshaped the treatment landscape for multiple advanced malignancies (1). However, their clinical benefits are accompanied by a distinct spectrum of immune-related adverse events (irAEs) that arise from immune dysregulation and may involve the skin, gastrointestinal tract, liver, lungs, endocrine organs, and virtually any other tissue (2). Because the severity of irAEs range from mild and self-limited toxicities to life-threatening or irreversible organ dysfunction, contemporary guidelines from the American Society of Clinical Oncology, Society for Immunotherapy of Cancer, and European Society for Medical Oncology emphasize early recognition, severity grading, and prompt organ-specific management (2–4).

Despite the maturation of irAE management frameworks, the substantial heterogeneity in toxicity susceptibility and phenotypes remains insufficiently understood. Biological sex is a plausible determinant for this variability. Sex chromosomes, gonadal hormones, and sex-biased immune gene regulation shape both innate and adaptive immunity, and females generally exhibit more vigorous humoral responses and higher baseline susceptibility to autoimmunity than males (5). These biological differences provide a strong mechanistic rationale for predicting sex-related variations in the frequency, organ distribution, and clinical course of irAEs during PD-1 blockade therapy.

However, the evidence for sex-specific irAE patterns remains inconsistent. While some studies have reported sex-related differences in selected organ-specific toxicities and clinical outcomes (6–12), others have observed no clear differences in the overall irAE burden (13, 14). This inconsistency may partly reflect differences in the study design. Many previous studies have evaluated ICIs as a single therapeutic class, despite well-recognized differences in the toxicity profiles and onset kinetics of anti-cytotoxic T-lymphocyte-associated protein 4 and anti-PD-1/programmed death ligand-1 therapies (15, 16). In addition, existing evidence has focused largely on event frequency, with comparatively little attention paid to toxicities. This temporal dimension is clinically relevant, as pooled clinical-trial data indicate that time to onset varies substantially across organ systems and according to the toxicity grade (15). Notably, a FAERS-based study by Chen et al. reported male-predominant signals for several immune-related toxicities, including cardiovascular, hepatic, pulmonary, musculoskeletal, nervous system, cutaneous, and renal toxicities, and found that males had a longer median time-to-onset (TTO) and a higher fatality rate than females (10). However, that study included all ICIs, rather than focusing specifically on PD-1 inhibitors. In addition, the analysis was performed at the System Organ Class (SOC) level and did not evaluate sex differences in individual irAEs.

Clarifying the sex-specific differences in irAE presentation may have practical implications for individualized toxicity surveillance and management. If certain toxicities are reported more frequently or occur earlier in the same sex, clinicians may be able to refine monitoring strategies, improve early recognition, and reduce treatment interruptions and serious outcomes. Such evidence could help support more personalized safety management of patients receiving PD-1 inhibitors.

Therefore, we conducted a sex-stratified pharmacovigilance analysis of irAEs associated with nivolumab and pembrolizumab using two large spontaneous reporting systems; the U.S. Food and Drug Administration Adverse Event Reporting System (FAERS) and Japanese Adverse Drug Event Report (JADER) databases. FAERS accepts global reports from manufacturers, healthcare professionals, and consumers, whereas JADER is a domestically managed Japanese database with more structured reports. We analyzed the databases separately to assess whether major sex-associated reporting patterns and onset-timing signals were directionally consistent across heterogeneous pharmacovigilance settings. By focusing specifically on nivolumab and pembrolizumab and incorporating both disproportionality and TTO analyses, the present study extends prior FAERS-based work and provides a more granular assessment of sex-specific irAE patterns during PD-1 inhibitor therapy. We hypothesized that sex would be associated with differential reporting patterns and TTO of selected irAEs following nivolumab and pembrolizumab therapy.

## 2 Methods

### 2.1 Study design and data sources

We conducted a retrospective pharmacovigilance study using two independent spontaneous reporting systems: the FAERS and JADER databases. The FAERS is the FDA post-marketing safety surveillance database for drugs and therapeutic biologics and includes reports submitted by manufacturers, healthcare professionals, and consumers. The FAERS data were obtained from the FDA Adverse Event Monitoring System public dashboard (https://fis.fda.gov/extensions/FPD-QDE-FAERS/FPD-QDE-FAERS.html). Public JADER files released by the Pharmaceuticals and Medical Devices Agency (https://www.info.pmda.go.jp/fukusayoudb/CsvDownload.jsp) comprise domestic adverse event reports submitted by marketing authorization holders in Japan. The study period was extended from the first quarter of 2014 to the second quarter of 2025 for both databases. As the FAERS and JADER differ in their regulatory context, database structure, coding practices, and reporting patterns, the two data sources were processed and analyzed separately rather than pooled.

### 2.2 Ethical statement

This study used publicly available, de-identified pharmacovigilance data, and therefore, did not require institutional review board approval or informed consent.

### 2.3 Data processing and case selection

Quarterly FAERS data files were imported into MySQL (version 14.14; Oracle Corporation, Redwood Shores, California, USA) for data management and preprocessing. Reports were deduplicated according to the FDA-recommended approach using case identifiers and follow-up versions such that only the most recent version of each case was retained. Cases listed in the quarterly deleted case files were excluded. Duplicate records within individual FAERS component tables were removed before database linkage, and the cleaned tables were subsequently merged using a report identifier to create an analysis-ready dataset.

The JADER data were also imported into MySQL for preprocessing and management. The component tables were merged using a report identifier to construct an integrated dataset. JADER data were processed independently of FAERS to preserve the original structure and reporting logic of each database.

**Figure 1:**
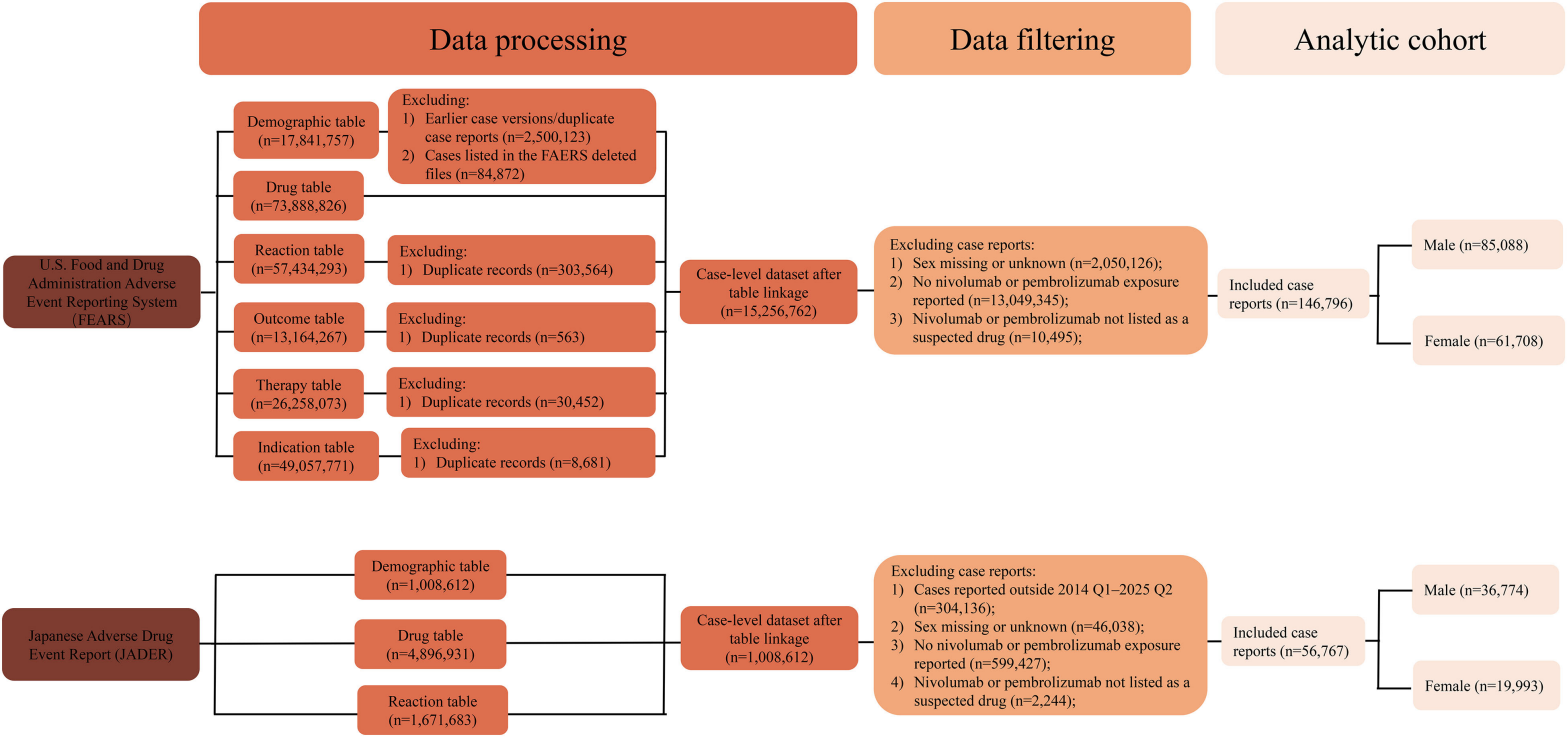
Flowchart of case selection and cohort construction in the FAERS and JADER databases.

To enhance cross-database comparability, a harmonized filtering strategy was applied to both databases. Eligible reports were required to meet the following criteria: (1) patient sex was reported and not coded as unknown, and (2) nivolumab or pembrolizumab was recorded as a suspected drug. In FAERS, this definition included both primary and secondary suspect reports, whereas in JADER, only a suspected drug is used. Reports meeting these criteria were included in the primary analyses. The overall workflow for data cleaning and case selection is shown in **Figure 1**. For TTO analyses, additional exclusion criteria were applied, including exclusion of reports with incomplete date information or non-positive TTO values (TTO ≤ 0; see Section 2.6).

### 2.4 Harmonization of primary neoplasm indications

Primary neoplasm indications recorded in the FAERS and JADER databases were harmonized according to the neoplasm chapter of the International Classification of Diseases, 10th Revision, and included malignant (C00-C97), *in situ* (D00-D09), and benign (D10-D36) neoplasms, as well as neoplasms of uncertain or unknown behavior (D37-D48). A unified mapping dictionary was applied to both databases to improve cross-database comparability.

### 2.5 Definition of irAEs

Adverse event terms from FAERS and JADER were standardized at the Preferred Term (PT) level using the Medical Dictionary for Regulatory Activities (version 28.0). Because the same underlying irAE may be reported under different but clinically related, overlapping, or synonymous PTs across reports, institutions, and countries, irAEs were not defined solely on the basis of reports explicitly labelled as “irAE.” Instead, we used a prespecified PT list covering clinically relevant immune-mediated toxicities, and clinically related PTs were harmonized into unified irAE categories to reduce outcome misclassification due to variable coding. In the primary analysis, a report was classified as containing an irAE if it included at least one PT, as listed in **Supplementary Table 1**, which provides a detailed mapping of each irAE category to its PTs.

### 2.6 Definition of TTO

TTO was defined as the interval (in days) between the initiation of the suspected PD-1 inhibitor and the onset of the reported adverse event and was calculated as follows:


TTO(days)=(event onset date−drug start date)+1

The TTO was calculated on a calendar-day basis. TTO analysis was performed on a subset of the primary study cohort. Only reports with complete day-level information for both the drug start date and adverse event onset date were eligible for TTO analysis. Reports were excluded if the calculated TTO was non-positive (TTO ≤ 0), indicating an implausible temporal sequence between drug exposure and event onset.

### 2.7 Statistical analysis

Categorical variables were summarized as frequencies and percentages, and between-group comparisons were performed using Pearson’s chi-square test. Continuous TTO data were summarized as medians and interquartile ranges (IQRs), and between-sex comparisons were conducted using the Wilcoxon rank-sum test. All statistical tests were two-sided, and a *p*-value < 0.05 was considered statistically significant.

Given the large number of endpoint-specific comparisons, *p*-values were adjusted for multiple testing using the Benjamini–Hochberg false discovery rate procedure. Multiplicity adjustment was applied separately within each analytic family, and the adjusted *p*-values were reported as *q*-values. Differences were considered statistically significant at a *q*-value of < 0.05.

#### 2.7.1 Disproportionality analysis

Sex-stratified disproportionality analyses were conducted separately on the FAERS and JADER databases using a two-by-two contingency tables framework. Crude reporting odds ratios (RORs) and 95% confidence intervals (CIs) were calculated to compare the odds of reporting each target irAE between female and male patients in reports involving nivolumab or pembrolizumab (**Supplementary Table 2**). A single report can contribute to more than one endpoint-specific analysis when multiple irAEs or SOCs have been reported. Each SOC and individual irAE was analyzed in a separate model as a binary endpoint (presence or absence within an eligible report). Thus, the same report could appear in multiple endpoint-specific models, but it was counted only once for each model.

To account for potential confounding factors, adjusted RORs (aRORs) were estimated using multivariate logistic regression models. For each irAE, the dependent variable was the presence or absence of the target event within the eligible report. Sex was the primary independent variable, with males as the reference category. The prespecified covariates included age, primary neoplasm category, PD-1 inhibitors (nivolumab or pembrolizumab), and concomitant therapy. For the FAERS, the reporting region was included as an adjustment variable. Missing values in categorical covariates were retained as separate categories, where applicable.

#### 2.7.2 TTO analysis

To further evaluate sex-specific differences in TTO while accounting for potential confounding factors, we fitted multivariable Weibull accelerated failure time (AFT) models for each irAE and estimated adjusted time ratios (aTRs) with 95% CIs, using males as the reference group. Covariate adjustment included age group, primary neoplasm category, PD-1 inhibitor (nivolumab or pembrolizumab), and concomitant therapy; reporting region was additionally included in the FAERS models. In this parameterization, an aTR < 1 indicated a shorter TTO in females than in males, whereas an aTR > 1 indicated a shorter TTO in males than in females.

The Weibull model was selected because it is widely used in pharmacovigilance TTO analyses and provides an interpretable parametric framework for characterizing event occurrences over time. In addition to the aTR, the model yields the Weibull scale parameter (α) and shape parameter (β). The shape parameter was used to descriptively characterize hazard patterns over time. An early failure profile was defined when β was < 1 and its 95% CI excluded 1 on the lower side; a random failure profile was defined when the 95% CI of β included 1; and a wear-out failure profile was defined when β was > 1 and its 95% CI excluded 1 on the upper side. Accordingly, early failure indicates that the hazard decreases over time and events tend to cluster in the earlier treatment period, random failure indicates an approximately constant hazard over time, and wear-out failure indicates an increasing hazard over time.

#### 2.7.3 Model adequacy for TTO analyses

Because no universally accepted sample size formula is available for multivariable Weibull AFT analyses of observational TTO data, the model adequacy was evaluated pragmatically using the events-per-parameter principle rather than a formal *a priori* sample size calculation. Using the conventional benchmark of at least 10 analyzable events per predictor parameter, the minimum required number of events was estimated to be 140 for FAERS, based on 14 prespecified parameters, and 100 for JADER, based on 10 prespecified parameters (17).

#### 2.7.4 Sensitivity and stratified analyses

We conducted sensitivity and stratification analyses to examine the robustness of the primary findings. In sensitivity analysis, we restricted the dataset to reports in which the event was explicitly designated as an irAE in the original record. A corresponding sensitivity analysis was not performed for TTO, because the number of reports explicitly identified as irAEs was too small to support stable modelling. In addition, because nivolumab and pembrolizumab differ in their approved indications and patterns of clinical use, drug-stratified analyses were conducted separately for each agent to evaluate whether the sex-specific reporting patterns and onset timing were consistent across the two PD-1 inhibitors.

## 3 Results

### 3.1 Sex-stratified report characteristics in FAERS and JADER

A total of 146,796 eligible PD-1 inhibitor-related adverse event reports were identified in the FAERS database, including 85,088 reports on males and 61,708 on females, and 56,767 eligible reports were identified in the JADER database, including 36,774 on males and 19,993 on females. The reported characteristics differed significantly according to sex in both databases (all *p* < 0.001). In both the FAERS and JADER databases, reports on females were relatively more common in the < 60-year age group, whereas reports on males were more frequently represented in the ≥ 60-year age group. The distribution of primary neoplasm categories also differed by sex; respiratory, intrathoracic, and gastrointestinal neoplasms were more common in reports on males, whereas genitourinary neoplasms were more frequently represented in reports on females. Pembrolizumab was mentioned at a greater frequency in female reports, whereas nivolumab was more frequently reported in male reports. Likewise, patterns of concomitant therapy differed significantly between the sexes (**Table 1**).

**Table 1:** Sex-stratified characteristics of adverse event reports for nivolumab and pembrolizumab in FAERS and JADER.

Characteristics	FAERS			JADER		
	Male (n = 85,088)	Female (n = 61,708)	*P*-value	Male (n = 36,774)	Female (n = 19,993)	*P*-value
Age group, years			<0.001			<0.001
< 60	17,929 (21.07%)	17,456 (28.29%)		5,809 (15.80%)	5,275 (26.38%)	
60–69	22,094 (25.97%)	13,942 (22.59%)		10,541 (28.66%)	5,082 (25.42%)	
≥ 70	29,636 (34.83%)	15,614 (25.30%)		18,734 (50.94%)	7,442 (37.22%)	
Unknown	15,429 (18.13%)	14,696 (23.82%)		1,690 (4.60%)	2,194 (10.97%)	
Primary neoplasm site			<0.001			<0.001
Respiratory and intrathoracic	26,049 (30.61%)	13,834 (22.42%)		15,606 (42.44%)	4,013 (20.07%)	
Genitourinary tract	17,266 (20.29%)	15,627 (25.32%)		9,881 (26.87%)	8,462 (42.32%)	
Skin (including melanoma)	11,996 (14.10%)	8,149 (13.21%)		2,176 (5.92%)	1,813 (9.07%)	
Gastrointestinal	9,919 (11.66%)	4,463 (7.23%)		5,756 (15.65%)	1,909 (9.55%)	
Other sites^†^	8,282 (9.73%)	10,808 (17.51%)		1,937 (5.27%)	3,103 (15.52%)	
Unspecified/missing	11,576 (13.60%)	8,827 (14.30%)		1,418 (3.86%)	693 (3.47%)	
PD-1 inhibitors			<0.001			<0.001
Nivolumab	47,632 (55.98%)	25,463 (41.26%)		21,931 (59.64%)	7,469 (37.36%)	
Pembrolizumab	37,456 (44.02%)	36,245 (58.74%)		14,843 (40.36%)	12,524 (62.64%)	
Concomitant therapy			<0.001			<0.001
Concomitant ipilimumab	14,440 (16.97%)	7,994 (12.95%)		10,828 (29.44%)	3,715 (18.58%)	
Concomitant TKI	9,670 (11.36%)	11,507 (18.65%)		2,508 (6.82%)	4,513 (22.57%)	
Reporting region			<0.001			
Asia	26,654 (31.33%)	15,722 (25.48%)		Not applicable		
Europe	25,236 (29.66%)	18,733 (30.36%)				
North America	28,351 (33.32%)	23,545 (38.16%)				
Other regions^§^	2,737 (3.22%)	1,962 (3.18%)				
Unknown	2,110 (2.48%)	1,746 (2.83%)				

FAERS, Food and Drug Administration Adverse Event Reporting System; JADER, Japanese Adverse Drug Event Report database; PD-1, programmed cell death-1; TKI, tyrosine kinase inhibitor.

Data are shown as counts and percentages and Pearson’s chi-square tests was used to compare males and females within each database. Statistical significance was defined as a two-sided *p*-value of < 0.05. Bold *p*-values indicate statistically significant differences.

^†^ Other sites include bone and soft tissue, breast, central nervous system, endocrine, head and neck, and lymphoid and hematopoietic tissues.

^§^ Other regions including Africa, South America, and Oceania.

### 3.2 Sex-stratified disproportionality signals at the SOC level

**Figure 2:**
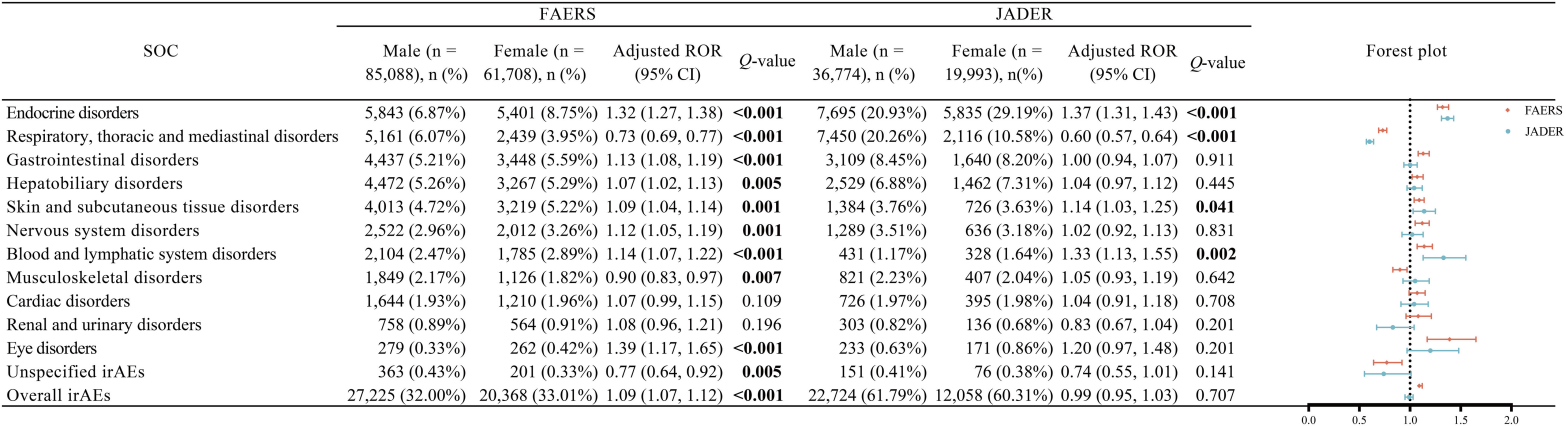
Forest plots of sex-stratified disproportionality signals at the SOC level for irAEs associated with nivolumab and pembrolizumab in FAERS and JADER. Models were adjusted for age group (< 60, 60–69, ≥ 70, and unknown), primary neoplasm site (respiratory and intrathoracic, genitourinary tract, skin, gastrointestinal, other sites, and unspecified/missing), PD-1 inhibitor (nivolumab or pembrolizumab), and concomitant therapy (ipilimumab or tyrosine kinase inhibitor); FAERS models were additionally adjusted for reporting region (Asia, Europe, North America, other regions, and unknown). Bold *q*-values indicate statistical differences. SOC, System Organ Class; irAEs, immune-related adverse events; FAERS, Food and Drug Administration Adverse Event Reporting System; JADER, Japanese Adverse Drug Event Report database; PD-1, programmed cell death-1; ROR, reporting odds ratio; 95% CI, 95% confidence interval.

At the SOC level, the most consistent sex-associated differences were endocrine, respiratory, thoracic, and mediastinal disorders. Endocrine disorders showed a female-predominant signal in both FAERS (aROR, 1.32; 95% CI, 1.27–1.38; *q* < 0.001) and JADER (aROR, 1.37; 95% CI, 1.31–1.43; *q* < 0.001). In contrast, respiratory, thoracic and mediastinal disorders showed a male-predominant pattern in both FAERS (aROR, 0.73; 95% CI, 0.69–0.77; *q* < 0.001) and JADER (aROR, 0.60; 95% CI, 0.57–0.64; *q* < 0.001). Consistent female-predominant signals were also observed for blood and lymphatic system disorders in both FAERS (aROR, 1.14; 95% CI, 1.07–1.22; *q* < 0.001) and JADER (aROR, 1.33; 95% CI, 1.13–1.55; *q* = 0.002). Skin and subcutaneous tissue disorders likewise showed modest but significant female-predominant associations in both databases (FAERS: aROR, 1.09; 95% CI, 1.04–1.14; *q* = 0.001; JADER: aROR, 1.14; 95% CI, 1.03–1.25; *q* = 0.041). Several additional SOC-level associations were observed in the FAERS group only (**Figure 2**).

Sensitivity and drug-stratified analyses yielded similar results. Across analyses, endocrine disorders remained consistently female predominant, whereas respiratory, thoracic, and mediastinal disorders remained consistently male predominant. However, some heterogeneity was observed between nivolumab and pembrolizumab treatments. In the nivolumab-stratified analyses, an additional consistent sex difference was observed in hepatobiliary disorders. In pembrolizumab-stratified analyses, additional sex-associated differences were identified for blood and lymphatic system disorders, eye disorders, and unspecified irAEs (**Supplementary Tables 3**, **4**).

### 3.3 Sex-stratified disproportionality signals at the individual irAE level

**Figure 3:**
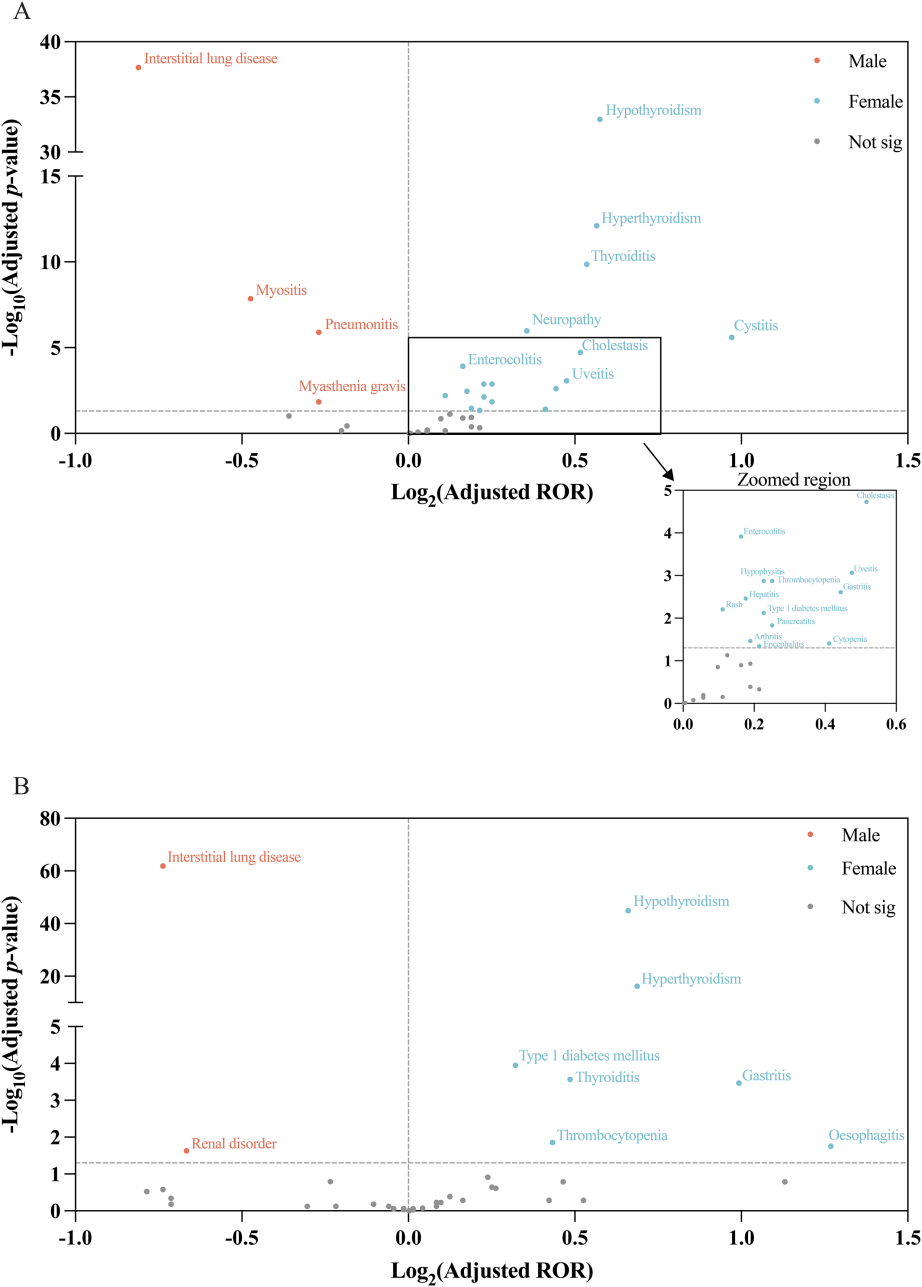
Sex-stratified differential reporting of irAEs associated with nivolumab and pembrolizumab. Volcano plots showing the association between sex and each irAE. Points to the right indicate irAEs reported more frequently in females, whereas points to the left indicate irAEs reported more frequently in males; non-significant signals are shown in grey. Selected irAEs with prominent sex differences are annotated. **(A)** FAERS. **(B)** JADER. irAEs, immune-related adverse events; FAERS, Food and Drug Administration Adverse Event Reporting System; JADER, Japanese Adverse Drug Event Report database; ROR, reporting odds ratio.

At the individual irAE level, a broadly consistent sex-specific pattern was observed across the FAERS and JADER databases. In both databases, hypothyroidism and hyperthyroidism emerged as the most prominent female-predominant signals, whereas interstitial lung disease showed the strongest male-predominant signal. Additional female-predominant signals consistently observed in both databases included thyroiditis, type 1 diabetes mellitus, gastritis, and thrombocytopenia (**Figure 3**).

Some database-specific differences were also noted. In FAERS, additional female-predominant associations were identified for cystitis, neuropathy, cholestasis, enterocolitis, uveitis, hypophysitis, hepatitis, rash, pancreatitis, arthritis, cytopenia, and encephalitis. FAERS also showed male-predominant associations for myositis, pneumonitis, and myasthenia gravis. In JADER, an additional female-predominant signal was observed for esophagitis, whereas renal disorder showed a modest but statistically significant male-predominant association (**Figure 3**).

These overall patterns were generally preserved in the sensitivity and drug-specific analyses, particularly the recurrent female-predominant thyroid-related signals and the male-predominant pulmonary signals (**Supplementary Figure 1**).

### 3.4 Sex-stratified TTO analysis at the SOC level

**Figure 4:**
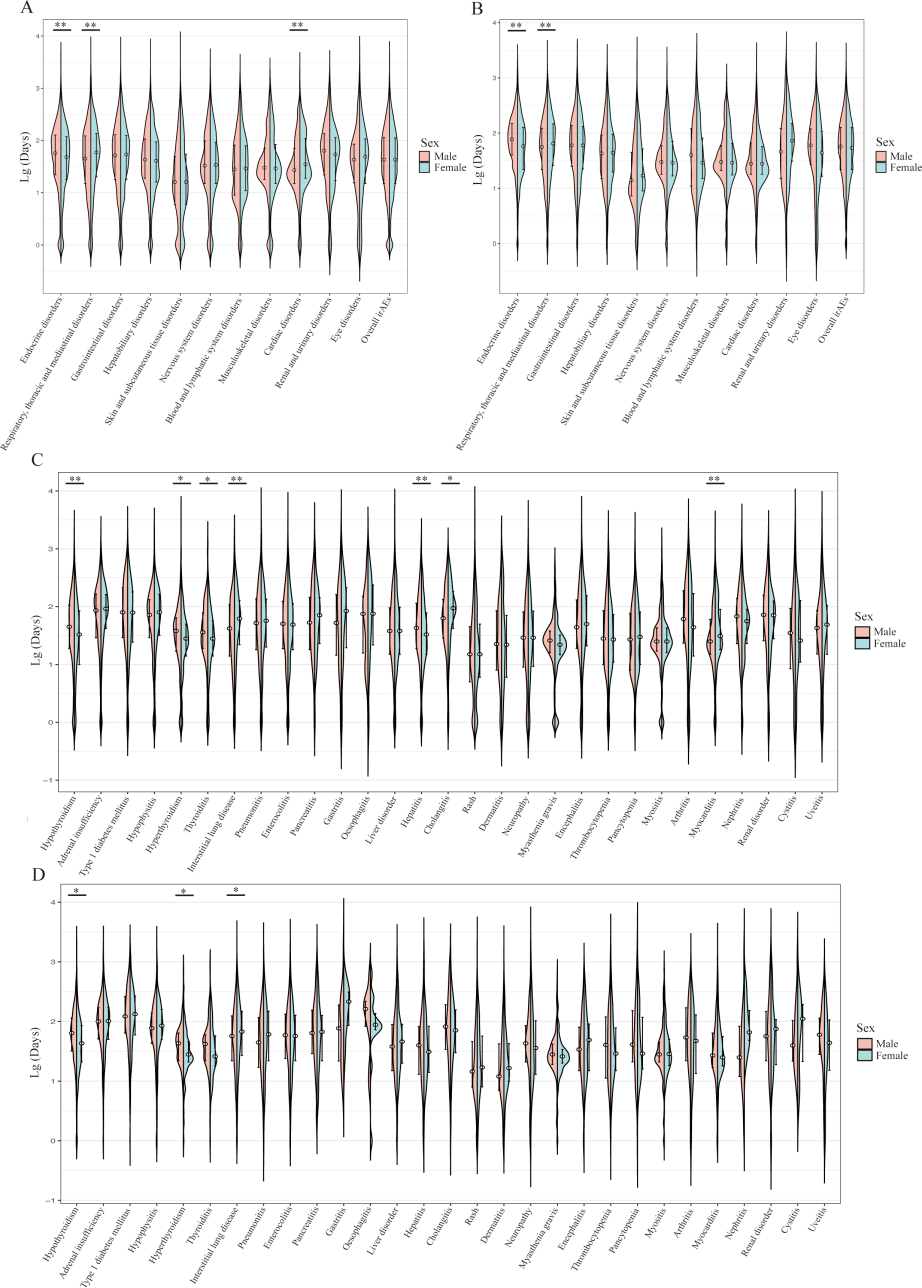
Sex-stratified TTO distributions of irAEs associated with PD-1 inhibitors in FAERS and JADER. **(A)** FAERS: TTO distributions of irAEs at the SOC level. **(B)** JADER: TTO distributions of irAEs at the SOC level. **(C)** FAERS: TTO distributions of individual irAE categories. **(D)** JADER: TTO distributions of individual irAE categories. TTO, time-to-onset; irAEs, immune-related adverse events; PD-1, programmed cell death-1; FAERS, Food and Drug Administration Adverse Event Reporting System; JADER, Japanese Adverse Drug Event Report database; SOC, System Organ Class. *q < 0.05; **q < 0.01.

Complete TTO data were available for 59,808 reports from the FAERS database and 25,396 reports from the JADER database. The characteristics of these reports are summarized in **Supplementary Table 5**, and comparisons between the reports included in and excluded from the TTO analyses are presented in **Supplementary Table 6**. At the SOC level, two patterns were observed across both databases. Respiratory, thoracic, and mediastinal disorders were associated with a shorter median TTO in males than in females (FAERS: 45 days [IQR, 15–122] vs. 59 days [IQR, 20–136], *q* < 0.01; JADER: 56 days [IQR, 22–123] vs. 65 days [IQR, 26–147], *q* < 0.01). Secondly, endocrine disorders showed an earlier median onset in females than in males (FAERS: 48 days [IQR, 18–119] vs. 57 days [IQR, 22–128], *q* < 0.01; JADER: 58 days [IQR, 22–127] vs. 77 days [IQR, 40–150], *q* < 0.01) (**Figures 4A, B**).

In multivariable Weibull AFT models, endocrine disorders remained significantly earlier in females in both FAERS (aTR, 0.90; 95% CI, 0.84–0.97; *q* = 0.039) and JADER (aTR, 0.88; 95% CI, 0.83–0.94; *q* < 0.001), with an early failure pattern in both databases. In FAERS only, females also showed earlier onset of hepatobiliary disorders (aTR, 0.90; 95% CI, 0.82–0.97; *q* = 0.039), whereas skin and subcutaneous tissue disorders occurred later in females than in males (aTR, 1.18; 95% CI, 1.04–1.33; *q* = 0.039) (**Table 2**).

**Table 2:** Sex-stratified Weibull AFT analysis of TTO for irAEs by SOC in FAERS and JADER.

SOC	FAERS							JADER						
	Sex	Number of cases	Scale parameter α (95% CI)	Shape parameter β (95% CI)	Hazard type	aTR (95% CI)	*Q*-value	Sex	Number of cases	Scale parameter α (95% CI)	Shape parameter β (95% CI)	Hazard type	aTR (95% CI)	*Q*-value
Endocrine disorders	Male	2,763	91.84 (87.28, 96.40)	0.79 (0.77, 0.81)	Early failure	Reference		Male	3,562	121.92 (117.50, 126.34)	0.96 (0.94, 0.98)	Early failure	Reference	
	Female	2,240	79.74 (75.26, 84.22)	0.78 (0.75, 0.80)	Early failure	0.90 (0.84, 0.97)	**0.039**	Female	2,299	96.95 (92.17, 101.73)	0.88 (0.85, 0.90)	Early failure	0.88 (0.83, 0.94)	**<0.001**
Respiratory, thoracic and mediastinal disorders	Male	2,675	80.57 (76.18, 84.97)	0.73 (0.71, 0.76)	Early failure	Reference		Male	4,045	89.98 (86.49, 93.47)	0.84 (0.82, 0.86)	Early failure	Reference	
	Female	1,056	97.38 (88.97, 105.79)	0.74 (0.70, 0.77)	Early failure	1.12 (1.02, 1.23)	0.061	Female	1,048	106.39 (98.58, 114.20)	0.87 (0.83, 0.91)	Early failure	1.05 (0.97, 1.14)	0.401
Gastrointestinal disorders	Male	2,124	92.13 (86.60, 97.67)	0.75 (0.72, 0.77)	Early failure	Reference		Male	1,494	104.41 (97.38, 111.44)	0.80 (0.77, 0.83)	Early failure	Reference	
	Female	1,521	91.39 (84.83, 97.95)	0.74 (0.71, 0.77)	Early failure	0.98 (0.89, 1.07)	0.719	Female	739	98.41 (89.50, 107.31)	0.84 (0.80, 0.89)	Early failure	0.91 (0.82, 1.02)	0.234
Hepatobiliary disorders	Male	2,229	80.17 (75.59, 84.75)	0.77 (0.75, 0.79)	Early failure	Reference		Male	1,257	73.57 (67.89, 79.25)	0.76 (0.73, 0.79)	Early failure	Reference	
	Female	1,586	72.83 (67.83, 77.82)	0.76 (0.73, 0.79)	Early failure	0.90 (0.82, 0.97)	**0.039**	Female	640	80.69 (72.41, 88.97)	0.80 (0.76, 0.85)	Early failure	1.06 (0.93, 1.20)	0.482
Skin and subcutaneous tissue disorders	Male	1,650	36.76 (33.78, 39.74)	0.63 (0.61, 0.65)	Early failure	Reference		Male	630	36.63 (32.03, 41.23)	0.66 (0.62, 0.70)	Early failure	Reference	
	Female	1,185	39.60 (35.60, 43.60)	0.60 (0.57, 0.62)	Early failure	1.18 (1.04, 1.33)	**0.039**	Female	342	38.70 (33.12, 44.27)	0.78 (0.72, 0.84)	Early failure	0.80 (0.66, 0.98)	0.149
Nervous system disorders	Male	1,021	65.20 (59.29, 71.11)	0.72 (0.68, 0.75)	Early failure	Reference		Male	600	56.58 (50.95, 62.21)	0.85 (0.80, 0.90)	Early failure	Reference	
	Female	738	67.84 (60.59, 75.08)	0.71 (0.68, 0.75)	Early failure	1.03 (0.90, 1.18)	0.719	Female	264	62.64 (52.92, 72.36)	0.83 (0.76, 0.90)	Early failure	1.12 (0.94, 1.33)	0.362
Blood and lymphatic system disorders	Male	1,128	58.26 (52.99, 63.54)	0.68 (0.65, 0.71)	Early failure	Reference		Male	224	80.94 (64.35, 97.53)	0.68 (0.61, 0.74)	Early failure	Reference	
	Female	893	58.35 (52.75, 63.95)	0.72 (0.69, 0.76)	Early failure	0.99 (0.87, 1.14)	0.919	Female	159	69.00 (54.83, 83.18)	0.80 (0.71, 0.89)	Early failure	0.86 (0.64, 1.15)	0.417
Musculoskeletal disorders	Male	742	63.27 (56.68, 69.87)	0.73 (0.69, 0.77)	Early failure	Reference		Male	431	60.55 (53.51, 67.59)	0.86 (0.80, 0.92)	Early failure	Reference	
	Female	454	66.69 (57.35, 76.03)	0.70 (0.65, 0.74)	Early failure	0.93 (0.79, 1.09)	0.537	Female	219	61.04 (51.54, 70.54)	0.90 (0.82, 0.99)	Early failure	0.94 (0.78, 1.15)	0.570
Cardiac disorders	Male	752	60.27 (54.07, 66.46)	0.74 (0.70, 0.78)	Early failure	Reference		Male	350	60.87 (52.23, 69.50)	0.78 (0.73, 0.84)	Early failure	Reference	
	Female	563	71.06 (63.65, 78.46)	0.84 (0.79, 0.89)	Early failure	0.92 (0.80, 1.06)	0.371	Female	174	52.30 (43.34, 61.26)	0.92 (0.83, 1.02)	Random failure	0.81 (0.65, 1.01)	0.184
Renal and urinary disorders	Male	311	101.84 (85.93, 117.76)	0.75 (0.69, 0.81)	Early failure	Reference		Male	119	96.93 (69.50, 124.36)	0.67 (0.59, 0.76)	Early failure	Reference	
	Female	203	79.62 (65.22, 94.02)	0.80 (0.72, 0.89)	Early failure	0.79 (0.63, 1.00)	0.116	Female	50	117.76 (78.94, 156.59)	0.89 (0.70, 1.08)	Random failure	1.17 (0.76, 1.81)	0.527
Overall irAEs	Male	12,721	78.98 (77.00, 80.96)	0.73 (0.72, 0.74)	Early failure	Reference		Male	10,968	95.93 (93.66, 98.21)	0.83 (0.82, 0.85)	Early failure	Reference	
	Female	8,650	77.36 (74.99, 79.73)	0.73 (0.71, 0.74)	Early failure	0.97 (0.94, 1.01)	0.300	Female	5,042	92.88 (89.68, 96.08)	0.85 (0.83, 0.87)	Early failure	0.96 (0.92, 1.00)	0.173

AFT, accelerated failure time, TTO, time-to-onset; irAEs, immune-related adverse events; SOC, system organ class; FAERS, Food and Drug Administration Adverse Event Reporting System; JADER, Japanese Adverse Drug Event Report database; aTR, adjusted time ratio; 95% CI, 95% confidence interval.

Models were adjusted for age group (< 60, 60–69, ≥ 70, and unknown), primary neoplasm site (respiratory and intrathoracic, genitourinary tract, skin, gastrointestinal, other sites, and unspecified/missing), PD-1 inhibitor (nivolumab or pembrolizumab), and concomitant therapy (ipilimumab or tyrosine kinase inhibitor); and FAERS models were additionally adjusted for reporting regions (Asia, Europe, North America, other regions, and unknown). Bold *q*-values indicate statistically significant differences.

In drug-stratified analyses, sex-associated differences in SOC-level TTO were more apparent among pembrolizumab reports. In this subgroup, an earlier TTO of endocrine and cardiac disorders in females was observed in both the databases. Other SOC-level TTO differences were database-specific (**Supplementary Table 7**).

### 3.5 Sex-stratified TTO analysis at the individual irAE level

At the individual irAE level, three events showed sex-associated differences in onset timing across both databases. Interstitial lung disease occurred earlier in males than in females in both the FAERS (42 days [IQR, 14–109] vs. 62 days [IQR, 22–128], *q* < 0.01) and JADER (57 days [IQR, 22–124] vs. 68 days [IQR, 27–148], *q* < 0.05). In contrast, hypothyroidism and hyperthyroidism each occurred earlier in females than in males across both systems. For hypothyroidism, the median TTO was 33 days (IQR, 10–85) in females vs. 45 days (IQR, 19–106) in males in the FAERS (*q* < 0.01), and 43 days (IQR, 21–85) vs. 64 days (IQR, 32–115), in the JADER (*q* < 0.05). For hyperthyroidism, the median TTO was 28 days (IQR, 14–50) in females vs. 38 days (IQR, 17–64) in males in the FAERS (*q* < 0.05), and 28 days (IQR, 21–46) vs. 43 days (IQR, 22–64), in the JADER (*q* < 0.05). Additional sex-associated differences in TTO were observed only in the FAERS database for several individual irAEs, including thyroiditis, hepatitis, cholangitis, and myocarditis (**Figures 4C, D**).

According to the adjusted Weibull AFT models, the TTO of hypothyroidism was significantly earlier in females in both databases (FAERS: aTR, 0.82; 95% CI, 0.72–0.93; *q* = 0.016; JADER: aTR, 0.79; 95% CI, 0.72–0.87; *q* < 0.001). Additional database-specific onset differences were identified for several other irAEs; however, these were less consistent across the FAERS and JADER databases (**Table 3**).

**Table 3:** Sex-stratified Weibull AFT analysis of TTO for individual irAEs in FAERS and JADER.

irAEs	FAERS							JADER						
	Sex	Number of cases	Scale parameter α (95% CI)	Shape parameter β (95% CI)	Hazard type	aTR (95% CI)	Q-value	Sex	Number of cases	Scale parameter α (95% CI)	Shape parameter β (95% CI)	Hazard type	aTR (95% CI)	Q-value
Hypothyroidism	Male	929	74.03 (67.69, 80.37)	0.79 (0.75, 0.83)	Early failure	Reference		Male	1,172	96.73 (90.89, 102.58)	1.00 (0.96, 1.04)	Random failure	Reference	
	Female	896	57.23 (51.63, 62.84)	0.71 (0.67, 0.74)	Early failure	0.82 (0.72, 0.93)	**0.016**	Female	1,072	65.92 (61.36, 70.48)	0.92 (0.88, 0.95)	Early failure	0.79 (0.72, 0.87)	**<0.001**
Adrenal insufficiency	Male	739	118.28 (107.90, 128.67)	0.86 (0.82, 0.91)	Early failure	Reference		Male	1,317	141.16 (133.54, 148.78)	1.06 (1.01, 1.10)	Wear-out failure	Reference	
	Female	497	128.38 (116.19, 140.57)	0.97 (0.91, 1.04)	Random failure	1.09 (0.95, 1.26)	0.501	Female	581	146.46 (134.46, 158.45)	1.05 (0.99, 1.11)	Random failure	1.10 (0.99, 1.21)	0.232
Type 1 diabetes mellitus	Male	372	136.37 (118.83, 153.92)	0.83 (0.77, 0.90)	Early failure	Reference		Male	595	198.48 (181.14, 215.82)	0.97 (0.91, 1.03)	Random failure	Reference	
	Female	266	122.46 (103.46, 141.45)	0.82 (0.74, 0.89)	Early failure	0.94 (0.78, 1.15)	0.776	Female	327	203.85 (179.33, 228.38)	0.95 (0.87, 1.03)	Random failure	1.00 (0.87, 1.15)	0.992
Hypophysitis	Male	499	103.61 (92.33, 114.89)	0.85 (0.79, 0.90)	Early failure	Reference		Male	491	113.24 (102.61, 123.87)	0.99 (0.93, 1.06)	Random failure	Reference	
	Female	341	106.04 (93.10, 118.97)	0.91 (0.84, 0.99)	Early failure	0.90 (0.77, 1.06)	0.501	Female	190	127.76 (108.94, 146.59)	1.02 (0.91, 1.12)	Random failure	1.07 (0.90, 1.26)	0.596
Hyperthyroidism	Male	464	54.75 (48.15, 61.36)	0.80 (0.75, 0.85)	Early failure	Reference		Male	492	57.50 (52.96, 62.05)	1.18 (1.11, 1.25)	Wear-out failure	Reference	
	Female	398	44.23 (38.81, 49.66)	0.85 (0.79, 0.91)	Early failure	0.75 (0.63, 0.89)	**0.009**	Female	393	44.90 (40.39, 49.41)	1.04 (0.97, 1.11)	Random failure	0.84 (0.74, 0.96)	0.070
Thyroiditis	Male	283	60.28 (51.75, 68.81)	0.87 (0.80, 0.94)	Early failure	Reference		Male	244	58.73 (51.11, 66.34)	1.03 (0.94, 1.11)	Random failure	Reference	
	Female	288	45.55 (38.94, 52.15)	0.84 (0.77, 0.91)	Early failure	0.70 (0.57, 0.87)	**0.009**	Female	187	52.73 (44.76, 60.69)	1.01 (0.90, 1.11)	Random failure	0.87 (0.71, 1.07)	0.345
Interstitial lung disease	Male	1,440	72.87 (67.45, 78.29)	0.73 (0.71, 0.76)	Early failure	Reference		Male	3,471	91.89 (88.05, 95.73)	0.84 (0.82, 0.86)	Early failure	Reference	
	Female	459	92.63 (81.89, 103.37)	0.83 (0.77, 0.89)	Early failure	1.01 (0.88, 1.16)	0.933	Female	900	108.01 (99.50, 116.51)	0.88 (0.83, 0.92)	Early failure	1.05 (0.96, 1.15)	0.478
Pneumonitis	Male	920	93.15 (84.86, 101.44)	0.77 (0.73, 0.80)	Early failure	Reference		Male	348	80.80 (70.14, 91.46)	0.84 (0.78, 0.91)	Early failure	Reference	
	Female	460	99.56 (85.46, 113.66)	0.68 (0.64, 0.73)	Early failure	1.18 (1.01, 1.38)	0.124	Female	93	99.98 (74.08, 125.88)	0.83 (0.70, 0.96)	Early failure	1.22 (0.91, 1.62)	0.345
Enterocolitis	Male	1,676	89.09 (83.09, 95.09)	0.75 (0.72, 0.78)	Early failure	Reference		Male	1,294	101.64 (94.31, 108.97)	0.80 (0.77, 0.83)	Early failure	Reference	
	Female	1,157	86.28 (79.13, 93.43)	0.74 (0.70, 0.77)	Early failure	0.95 (0.86, 1.06)	0.628	Female	641	93.67 (84.66, 102.68)	0.85 (0.80, 0.90)	Early failure	0.89 (0.79, 1.00)	0.232
Pancreatitis	Male	248	106.00 (87.25, 124.74)	0.74 (0.68, 0.81)	Early failure	Reference		Male	181	120.41 (97.95, 142.87)	0.83 (0.74, 0.92)	Early failure	Reference	
	Female	217	108.45 (89.27, 127.63)	0.80 (0.72, 0.87)	Early failure	0.97 (0.76, 1.25)	0.934	Female	84	107.74 (77.81, 137.67)	0.81 (0.68, 0.95)	Early failure	0.95 (0.68, 1.34)	0.812
Liver disorder	Male	1,023	70.55 (64.38, 76.71)	0.74 (0.71, 0.78)	Early failure	Reference		Male	907	70.85 (64.45, 77.25)	0.76 (0.73, 0.80)	Early failure	Reference	
	Female	687	74.62 (66.19, 83.06)	0.70 (0.66, 0.74)	Early failure	1.03 (0.90, 1.18)	0.776	Female	449	81.28 (71.56, 91.01)	0.82 (0.77, 0.88)	Early failure	1.07 (0.92, 1.23)	0.525
Hepatitis	Male	820	81.49 (73.74, 89.25)	0.76 (0.72, 0.80)	Early failure	Reference		Male	258	61.61 (51.25, 71.98)	0.77 (0.70, 0.84)	Early failure	Reference	
	Female	621	62.18 (55.76, 68.60)	0.81 (0.76, 0.85)	Early failure	0.74 (0.65, 0.85)	**0.001**	Female	138	69.57 (52.42, 86.72)	0.72 (0.64, 0.80)	Early failure	1.26 (0.93, 1.71)	0.303
Cholangitis	Male	245	105.03 (89.41, 120.66)	0.89 (0.81, 0.97)	Early failure	Reference		Male	125	145.38 (111.41, 179.36)	0.79 (0.69, 0.90)	Early failure	Reference	
	Female	150	134.77 (111.67, 157.86)	0.98 (0.86, 1.11)	Random failure	1.09 (0.86, 1.39)	0.666	Female	67	111.98 (82.01, 141.96)	0.94 (0.77, 1.12)	Random failure	0.72 (0.50, 1.05)	0.232
Rash	Male	1,416	34.46 (31.41, 37.50)	0.63 (0.60, 0.65)	Early failure	Reference		Male	436	37.24 (31.62, 42.85)	0.66 (0.62, 0.70)	Early failure	Reference	
	Female	1,037	36.80 (32.83, 40.77)	0.60 (0.57, 0.62)	Early failure	1.15 (1.01, 1.31)	0.124	Female	227	39.40 (32.26, 46.55)	0.76 (0.69, 0.84)	Early failure	0.86 (0.68, 1.09)	0.377
Dermatitis	Male	258	50.27 (40.72, 59.81)	0.68 (0.62, 0.74)	Early failure	Reference		Male	204	34.05 (26.50, 41.60)	0.66 (0.59, 0.72)	Early failure	Reference	
	Female	168	55.52 (40.66, 70.38)	0.60 (0.53, 0.67)	Early failure	1.13 (0.83, 1.52)	0.666	Female	118	36.20 (27.69, 44.71)	0.82 (0.71, 0.92)	Early failure	0.72 (0.50, 1.04)	0.232
Neuropathy	Male	345	54.77 (45.59, 63.95)	0.67 (0.61, 0.72)	Early failure	Reference		Male	137	69.20 (53.88, 84.53)	0.80 (0.70, 0.90)	Early failure	Reference	
	Female	298	55.72 (45.74, 65.70)	0.67 (0.61, 0.73)	Early failure	1.31 (1.02, 1.69)	0.124	Female	55	80.14 (47.31, 112.96)	0.68 (0.55, 0.82)	Early failure	1.20 (0.82, 1.77)	0.506
Myasthenia gravis	Male	252	36.60 (31.57, 41.64)	0.95 (0.87, 1.03)	Random failure	Reference		Male	272	43.22 (37.67, 48.76)	0.98 (0.91, 1.06)	Random failure	Reference	
	Female	120	33.56 (26.67, 40.45)	0.92 (0.80, 1.04)	Random failure	0.88 (0.70, 1.10)	0.582	Female	113	36.72 (31.34, 42.10)	1.34 (1.17, 1.50)	Wear-out failure	0.76 (0.61, 0.93)	0.070
Encephalitis	Male	246	86.49 (70.49, 102.49)	0.71 (0.65, 0.78)	Early failure	Reference		Male	157	62.33 (49.51, 75.16)	0.80 (0.71, 0.90)	Early failure	Reference	
	Female	177	95.47 (76.15, 114.79)	0.77 (0.68, 0.85)	Early failure	1.07 (0.82, 1.41)	0.776	Female	83	80.47 (59.74, 101.19)	0.89 (0.74, 1.03)	Random failure	1.12 (0.81, 1.54)	0.613
Thrombocytopenia	Male	666	60.77 (53.70, 67.85)	0.69 (0.65, 0.73)	Early failure	Reference		Male	150	80.47 (60.26, 100.68)	0.68 (0.60, 0.75)	Early failure	Reference	
	Female	541	54.98 (47.99, 61.97)	0.70 (0.66, 0.75)	Early failure	0.99 (0.83, 1.18)	0.934	Female	107	65.30 (49.08, 81.53)	0.81 (0.70, 0.92)	Early failure	0.93 (0.61, 1.40)	0.812
Pancytopenia	Male	400	49.58 (42.50, 56.65)	0.73 (0.68, 0.78)	Early failure	Reference		Male	54	102.28 (59.82, 144.75)	0.68 (0.55, 0.81)	Early failure	Reference	
	Female	311	57.56 (49.08, 66.03)	0.80 (0.73, 0.87)	Early failure	1.02 (0.82, 1.26)	0.934	Female	47	80.12 (49.16, 111.09)	0.79 (0.62, 0.95)	Early failure	0.62 (0.39, 1.00)	0.070
Myositis	Male	484	41.65 (37.24, 46.06)	0.89 (0.84, 0.95)	Early failure	Reference		Male	326	49.90 (43.58, 56.22)	0.91 (0.84, 0.98)	Early failure	Reference	
	Female	242	45.32 (37.82, 52.83)	0.81 (0.74, 0.88)	Early failure	1.11 (0.92, 1.34)	0.582	Female	150	53.19 (43.77, 62.61)	0.96 (0.86, 1.07)	Random failure	1.04 (0.83, 1.29)	0.812
Arthritis	Male	262	115.91 (95.09, 136.73)	0.71 (0.65, 0.78)	Early failure	Reference		Male	109	96.64 (74.05, 119.22)	0.85 (0.72, 0.97)	Early failure	Reference	
	Female	214	97.65 (76.81, 118.50)	0.66 (0.59, 0.73)	Early failure	0.74 (0.57, 0.96)	0.110	Female	70	77.45 (54.22, 100.69)	0.82 (0.67, 0.98)	Early failure	0.68 (0.46, 1.02)	0.232
Myocarditis	Male	671	53.66 (47.94, 59.39)	0.75 (0.71, 0.79)	Early failure	Reference		Male	326	57.29 (48.83, 65.76)	0.78 (0.72, 0.84)	Early failure	Reference	
	Female	510	66.52 (59.24, 73.79)	0.84 (0.79, 0.89)	Early failure	0.95 (0.81, 1.10)	0.666	Female	169	51.93 (42.88, 60.98)	0.92 (0.83, 1.01)	Random failure	0.84 (0.66, 1.05)	0.303

AFT, accelerated failure time, TTO, time-to-onset; irAEs, immune-related adverse events; FAERS, Food and Drug Administration Adverse Event Reporting System; JADER, Japanese Adverse Drug Event Report database; aTR, adjusted time ratio; 95% CI, 95% confidence interval.

Models were adjusted for age group (< 60, 60–69, ≥ 70, and unknown), primary neoplasm site (respiratory and intrathoracic, genitourinary tract, skin, gastrointestinal, other sites, and unspecified/missing), PD-1 inhibitor (nivolumab or pembrolizumab), and concomitant therapy (ipilimumab or tyrosine kinase inhibitor), and FAERS models were additionally adjusted for reporting regions (Asia, Europe, North America, other regions, and unknown). Bold *q*-values indicate statistically significant differences.

Drug-stratified analyses further showed that, among pembrolizumab reports, hypothyroidism occurred earlier in females, whereas sex-associated differences for other irAEs were less consistent across the databases (**Supplementary Table 8**).

## 4 Discussion

In this dual-database pharmacovigilance study, spanning the first quarter of 2014 to the second quarter of 2025, we identified an organ-specific pattern of sex-associated irAEs associated reporting with nivolumab and pembrolizumab across the FAERS and JADER databases. The most consistent finding was a female predominant signal for endocrine toxicities, particularly thyroid-related events, alongside a male predominant signal for respiratory toxicities, particularly interstitial lung disease. Incorporating TTO analyses provided an important temporal dimension to these differences: hypothyroidism tended to occur earlier in females. Although several additional associations were observed in only one database or drug stratum, the most robust findings were female predominance and earlier onset of thyroid toxicities, and male predominance of pulmonary toxicities.

A previous FAERS-based study by Chen et al. reported male predominant signals for cardiovascular, hepatitis, lung, musculoskeletal, nervous system, skin, and renal toxicities, alongside a longer median TTO and higher fatality rate in males than in females (10). In contrast, in our SOC-level analyses, respiratory, thoracic, and mediastinal, and musculoskeletal disorders were predominantly in males, whereas endocrine, blood, lymphatic system, and skin and subcutaneous tissue disorders were predominantly in females across both databases. Several factors could explain these differences. First, Chen et al. included all the ICI classes, whereas our analysis was restricted to the nivolumab and pembrolizumab classes. Second, they compared males and females in a 1:1 matched FAERS sample, whereas we analyzed the fully eligible cohort with multivariable adjustment. In addition, even at the SOC level, the overall pattern may shift depending on the underlying composition of individual PTs and irAEs within each SOC. However, regarding TTO, an earlier FAERS study reported a longer median onset in males only in the PD-1 subgroup, which is directionally consistent with our findings and may reflect the greater clinical homogeneity of PD-1-restricted analyses.

A comparison with broader literature suggests that sex-related variations in irAEs may be more readily detected in specific organ systems than in the overall irAE burden (6–14). Several previous studies, including a large cohort analysis by Jing et al. and the MOUSEION-07 systematic review and meta-analysis, found no stable sex difference in overall irAEs (13, 14). Consistent with these reports, we did not observe a uniform sex difference in overall irAEs or across all SOC-level categories. This may reflect an important limitation of composite endpoints, which combine heterogeneous irAEs into a single outcome, which risks concealing organ-specific signals that vary in direction and magnitude. In the present study, the strongest female-predominant signals involved endocrine and thyroid-related toxicities, whereas the most prominent male-predominant signals involved pulmonary toxicities, particularly interstitial lung disease.

The endocrine findings of the present study are broadly consistent with those of prior thyroid-focused pharmacovigilance and clinical studies. Kennedy et al. and Muir et al. reported that female sex was independently associated with hypothyroidism during anti-PD-1 therapy (18, 19), whereas Lu et al. showed that ICI-related new-onset thyroid dysfunction, including within anti-PD-1 subgroups, was more prominent in females (20). Earlier onset of thyroid dysfunction in female patients has also been described previously, including earlier thyrotoxicosis onset in females than in males (9). In this analysis, these observations were refined in two ways. First, the female-predominant endocrine signal remained directionally consistent across the FAERS and JADER databases at the SOC level. Second, at the irAE level, this broader endocrine pattern appeared to be driven mainly by thyroid-related toxicities, particularly hypothyroidism and hyperthyroidism. Importantly, the temporal dimension was also consistent: endocrine disorders occurred earlier in females in both databases and hypothyroidism remained earlier in females in the adjusted Weibull models across both systems. Taken together, these observations suggest that thyroid-related irAEs are among the most consistent sex-associated safety signals during nivolumab and pembrolizumab therapy. These findings may be clinically relevant because the timing of toxicity directly influences surveillance windows and the scheduling of laboratory or imaging assessments.

By contrast, the pulmonary findings are directionally consistent with some, but not all, prior studies. In our analysis, respiratory, thoracic, and mediastinal disorders were predominantly in males at the SOC level, and interstitial lung disease was the strongest male predominant signal at the irAE level. This pattern is consistent with the findings of Yamaguchi et al. and Zhou et al., who identified male sex and pre-existing pulmonary vulnerability as relevant risk factors for checkpoint inhibitor pneumonitis (21, 22). However, not all cohort studies reported similar results. For example, Duma et al., reported more cases of pneumonitis among females within NSCLC treated with anti-PD-1 therapy (23). Further studies are required to clarify the causes of this heterogeneity.

Several biological and clinical mechanisms may have plausibly contributed to the sex-associated patterns observed in this study, although these cannot be established from spontaneously reported data. The female predominance of thyroid irAEs is biologically plausible considering the well-recognized female bias in autoimmune thyroid disease and the broader female predisposition to autoimmunity. Estrogen may enhance humoral and B cell-mediated immune responses, and some immune-related genes on the X chromosome may escape inactivation, thereby amplifying inflammatory signaling (24, 25). Baseline thyroid autoantibodies may lower the threshold for thyroid dysfunction when PD-1-mediated immune tolerance is disrupted (26). In contrast, the male predominance of pulmonary signals may reflect a different background context, including higher cumulative exposure to smoking, chronic lung injury, interstitial lung abnormalities, or prior thoracic radiotherapy, in some tumors (22, 27). These factors create a pulmonary microenvironment that is more vulnerable to immune-mediated injuries.

Although mechanisms related to sex hormones, immune regulation, or comorbidity patterns may be relevant, these explanations remain speculative in the absence of prospectively collected clinical data. Accordingly, our findings are interpreted as identifying sex-associated safety signals that may warrant further validation in well-characterized cohorts. From a clinical perspective, sex may be a clinically relevant dimension for refining surveillance strategies during PD-1 inhibitor therapy. In particular, closer monitoring of thyroid function during the early treatment period may be especially relevant in female patients, whereas male patients, particularly those with pulmonary risk factors, may benefit from heightened vigilance for respiratory symptoms and early radiographic evaluation when clinically indicated. These observations may provide more individualized toxicity monitoring and patient counselling.

Drug-stratified analyses also require cautious interpretation. Overall, the main endocrine and pulmonary patterns were preserved across the stratified analyses; however, sex-associated differences appeared more pronounced and somewhat broader among the pembrolizumab reports. However, these subgroup differences should not be interpreted as drug-specific. In terms of the baseline characteristics, pembrolizumab was more frequently associated with female reports, whereas nivolumab was more frequently associated with male reports, suggesting differences in drug utilization, tumor-type distribution, concomitant therapy, and possibly monitoring or reporting intensity between the two drugs. Thus, the stronger sex-associated signals observed in pembrolizumab-stratified analyses may reflect a combination of true clinical heterogeneity and reporting-related factors.

Another point that warrants emphasis is that the FAERS and JADER databases are structurally different spontaneous reporting systems, which may have influenced the observed patterns. The FAERS accepts reports from manufacturers, healthcare professionals, and consumers across multiple regions, whereas the JADER is a domestically managed reporting system with a different reporting structure and curation framework. Accordingly, the cross-database comparison in this study should not be interpreted as a direct head-to-head validation between interchangeable systems. Instead, we treated the FAERS and JADER databases as two heterogeneous pharmacovigilance settings and examined whether the major sex-associated signals were directionally consistent across them. Concordant findings may strengthen confidence that certain signals are not unique to a single reporting environment; however, they do not exclude the possibility that shared, or system-specific reporting biases contributed to the observed associations.

This study had several limitations. First, both the FAERS and JADER databases are spontaneous reporting systems; therefore, the findings should be interpreted as signal-generating rather than causal. The inclusion of an adverse event report in either database does not mean that the reported event was directly caused by nivolumab or pembrolizumab, and no causal relationship could be confirmed based on spontaneous reports alone. Second, both databases are subject to important data quality limitations, including underreporting, possible residual duplicate reporting, missing data, incomplete clinical information, variable report quality, and inconsistencies in coding and documentation. Third, the FAERS and JADER databases are structurally different reporting systems with different sources, coding practices, curation frameworks, and regulatory contexts. These differences may influence the detection, magnitude, and direction of sex-associated reporting signals. Moreover, the differences between the two databases in population characteristics, genetic background, disease distribution, treatment practices, and reporting behavior cannot be fully accounted for. Therefore, the similarities between the two systems should not be interpreted as proof of true biological reproducibility. Rather, directional consistency across FAERS and JADER should only be viewed as supportive of signal robustness across heterogeneous pharmacovigilance settings. Fourth, substantial residual confounding factors are likely. Although we performed multivariate adjustment, many clinically relevant variables were unavailable, incomplete, or inconsistently coded, including smoking history, baseline comorbidities, prior radiotherapy, tumor stage, performance status, and detailed treatment history. This issue is particularly important in oncology, where patients commonly receive multiple concomitant medications and multimodal anticancer therapies, making drug-event attribution inherently uncertain. In addition, our exposure definition was based on suspected drug designation rather than a restriction to primary suspect reports. Although this approach improved harmonization between the FAERS and JADER databases, the inclusion of secondary suspect reports in the FAERS may have introduced additional confounding factors related to less certain attributions and more complex co-medication settings. Finally, the TTO analyses were restricted to reports with complete and valid data, and the reports included in the TTO analyses differed systematically from those not included, suggesting selection bias.

In conclusion, this dual-database analysis suggests that nivolumab- and pembrolizumab-associated irAEs show directionally consistent sex-associated patterns across the FAERS and JADER, most notably female-predominant and earlier thyroid toxicities, and male-predominant pulmonary toxicities, particularly interstitial lung disease. These observations should be interpreted as hypothesis-generating rather than causal; however, they support the possibility that sex may be a clinically relevant dimension for refining toxicity surveillance during PD-1 inhibitor therapy. Future studies should further validate these findings in well-characterized prospective cohorts and clarify the underlying mechanisms through the integration of clinical risk factors, imaging, auto-antibody profiles, and other immune biomarkers.

## Data Availability

Publicly available datasets were analyzed in this study. This data can be found here: FAERS data were obtained from the FDA Adverse Event Monitoring System public dashboard (https://fis.fda.gov/extensions/FPD-QDE-FAERS/FPD-QDE-FAERS.html). The public JADER files, released by the Pharmaceuticals and Medical Devices Agency (https://www.info.pmda.go.jp/fukusayoudb/CsvDownload.jsp).
