# Supplementary material for: Sex-specific reporting patterns and onset timing of immune-related adverse events associated with nivolumab and pembrolizumab: a dual-database pharmacovigilance analysis
Source: Front Immunol. 2026 May 13;17:1837640. doi: 10.3389/fimmu.2026.1837640 (PMC13212199; doi:10.3389/fimmu.2026.1837640)
Supplement: Supplementary file 1 [file Image1.pdf]

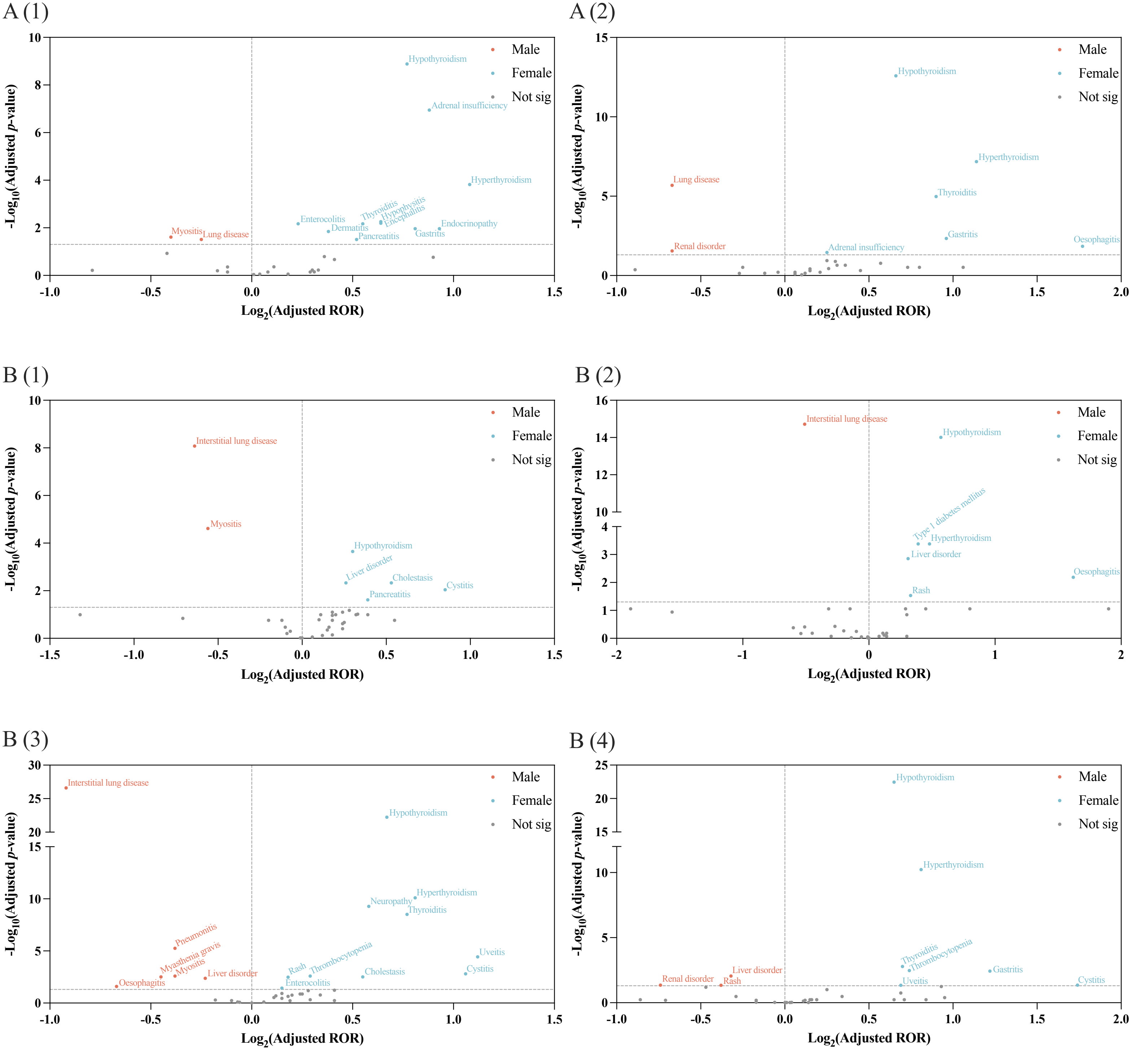

Supplementary Figure S1. Sensitivity and drug-specific analyses of sex-stratified differential reporting of irAEs associated with nivolumab and pembrolizumab. Volcano plots showing the association between sex and each irAE. Points to the right indicate irAEs reported more frequently in females, whereas points to the left indicate irAEs reported more frequently in males; non-significant signals are shown in grey. Selected irAEs with prominent sex differences are annotated. (A) Sensitivity analysis restricted to reports explicitly designated as irAEs: (1) FAERS; (2) JADER. (B) Drug-specific analysis: nivolumab in (1) FAERS and (2) JADER, and pembrolizumab in (3) FAERS and (4) JADER. irAEs, immune-related adverse events; FAERS, Food and Drug Administration Adverse Event Reporting System; JADER, Japanese Adverse Drug Event Report database; ROR, reporting odds ratio.
